# Application of a novel regulatable Cre recombinase system to define the role of liver and gut metabolism in drug oral bioavailability

**DOI:** 10.1042/BJ20140582

**Published:** 2015-02-01

**Authors:** Colin J. Henderson, Lesley A. McLaughlin, Maria Osuna-Cabello, Malcolm Taylor, Ian Gilbert, Aileen W. McLaren, C. Roland Wolf

**Affiliations:** * Division of Cancer Research, Level 9, Jacqui Wood Cancer Centre, University of Dundee, Ninewells Hospital & Medical School, Dundee DD1 9SY, U.K.; † Drug Discovery Unit, College of Life Sciences, University of Dundee, Dow Street, Dundee DD1 5EH, U.K.

**Keywords:** cytochrome P450 oxidoreductase, conditional inducible deletion, drug bioavailability, liver, gut

## Abstract

The relative contribution of hepatic compared with intestinal oxidative metabolism is a crucial factor in drug oral bioavailability and therapeutic efficacy. Oxidative metabolism is mediated by the cytochrome P450 mono-oxygenase system to which cytochrome P450 reductase (POR) is the essential electron donor. In order to study the relative importance of these pathways in drug disposition, we have generated a novel mouse line where Cre recombinase is driven off the endogenous Cyp1a1 gene promoter; this line was then crossed on to a floxed POR mouse. A 40 mg/kg dose of the Cyp1a1 inducer 3-methylcholanthrene (3MC) eliminated POR expression in both liver and small intestine, whereas treatment at 4 mg/kg led to a more targeted deletion in the liver. Using this approach, we have studied the pharmacokinetics of three probe drugs – paroxetine, midazolam, nelfinavir – and show that intestinal metabolism is a determinant of oral bioavailability for the two latter compounds. The Endogenous Reductase Locus (ERL) mouse represents a significant advance on previous POR deletion models as it allows direct comparison of hepatic and intestinal effects on drug and xenobiotic clearance using lower doses of a single Cre inducing agent, and in addition minimizes any cytotoxic effects, which may compromise interpretation of the experimental data.

## Introduction

Cytochrome P450 reductase (NADPH-cytochrome P450 oxidoreductase; EC 1.6.2.4; abbreviated as either POR or CPR) is the key electron donor to the cytochrome P450 (P450) superfamily of xenobiotic metabolizing enzymes. It also plays a number of important roles in endogenous metabolism, passing electrons to a range of acceptors including cytochrome *b*_5_ (supporting fatty acid desaturase and elongase activities), squalene monooxygenase (sterol biosynthesis) and haem oxygenase (haem degradation). It can also directly mediate the activation of bioreductive prodrugs such as mitomycin C and tirapazamine [1].

One of the most important factors determining the therapeutic utility of drugs is their bioavailability, which is often determined by P450-mediated hepatic and extra-hepatic metabolism. Accurate prediction of drug clearance and the contribution of cytochrome P450s (P450s) to this process are therefore critical for the progression of new chemical entities through the preclinical stage of drug development [2,3]. The poor bioavailability of many drugs, including chemotherapeutic agents, often means that administration has to be carried out intravenously. In order to optimize oral drug delivery, it is essential to characterize the contribution of the gut to first pass metabolism. However, the obvious approach (that of knocking out all the P450s from specific tissues) is impractical because of the large number of P450 isoforms, as well as their individual genetic variability.

In theory, the most effective way of creating a transgenic mouse which lacks any P450 activity would be to knock out the POR gene, since this electron donor is essential to the function of all P450s. However, germline deletion of this gene leads to embryonic lethality and no offspring survive to birth [4–6]. An alternative approach, which permits the generation of live mice lacking POR activity at key sites, is to engineer a tissue-specific deletion via either constitutive or induced expression driven by an appropriately targeted promoter. Two such hepatic POR-null lines, our Hepatic Reductase Null (HRN™) and the liver Cpr-null, were reported in 2003 [7–9]. Both took advantage of the Cre-lox system to delete POR from the liver and leave it intact in other tissues: in order to achieve this, mice with a floxed POR gene (*Por*^lox/lox^) were crossed with mice in which Cre recombinase expression was driven by the albumin promoter (Alb-Cre). Mice with such a hepatic deletion of POR can be used to establish the role of the hepatic P450 system in pharmacodynamic and toxicological responses to drugs over both acute and chronic timescales [7,10–15].

The phenotype observed in the original POR-null mouse line [7–9], the existence of human syndromes characterized by polymorphisms or genetic defects in POR [1] and the putative role of POR in various aspects of systemic toxicity including pulmonary and mammary carcinogenesis have stimulated various investigators to develop specialized tissue-specific POR-null mouse lines for the study of these processes. Most of these have been created by crossing *Por*^lox/lox^ mice with lines in which Cre expression is driven by a tissue-specific promoter. Models used to study pharmacological and toxicological processes have included a cardiomyocyte-specific model in which Cre is driven by the *α*-myosin heavy chain promoter (used to study doxorubicin-induced cardiotoxicity [16]), a brain neuron-specific model driven by the Camk2a promoter (used to study the role of POR in nociception [17]), an intestine-specific model using the Villin promotor (to study nifedipine metabolism [18]) and a mammary gland-specific model driven by the mouse mammary tumour virus promoter (used to study dimethyl benzanthracene adduct formation in mammary tissue [19]).

The limitation of these models is that they do not allow POR expression in the target tissue to be manipulated in real time. In contrast, inducible models allow POR expression to be switched off at a chosen moment during development or in adult life. More readily manipulable inducible POR-null models can be created by taking advantage of the tight regulation of gene expression in response to compounds which act via the aryl hydrocarbon receptor (AhR). We established a mouse line (Reductase Conditional Null, RCN) in which POR can be deleted conditionally via the action of this receptor [20]. Our model was generated by crossing *Por*^lox/lox^ mice with animals in which the expression of Cre is conditionally regulated by the rat CYP1A1 promoter, providing a tightly regulated method of controlling its expression *in vivo* [21–23]. Using this model, we were able to achieve either knockdown of POR expression in the liver alone or in both the liver and small intestine, depending on the dosing schedule of the Cyp1a1 inducers *β*-naphthoflavone (*β*NF) and 3-methylcholanthrene (3MC). Following induction, this model had essentially the same hepatic phenotype as the HRN model, including elevated P450 expression and hepatic steatosis coupled with minimal hepatic P450 activity. The utility of this model has been demonstrated by means of an experiment in which the mice were dosed orally with the colorectal carcinogen 2-amino-1-methyl-6-phenylimidazo[4,5-*b*]pyridine (PhIP) 2 weeks after 3MC treatment [24]. Following this regime, adduct formation in the colon was reduced by 50% in mice which had received both 3MC and PhIP compared with those which had received PhIP alone, demonstrating the key role of hepatic P450-mediated metabolic activation in the formation of colonic PhIP-DNA adducts.

The RCN model has obvious advantages over one in which hepatic or intestinal POR is deleted by a strong constitutive tissue-specific promoter such as those which regulate albumin or villin expression; however, it is not without its limitations. In particular, it was engineered by random transgenesis, so the site of integration of the CYP1A1 promoter driving Cre recombinase expression is unknown and may not be optimal. Furthermore, relatively high doses of two different inducers were required to achieve liver or liver and gut deletion of POR. We have now therefore created a more sophisticated model by generating a mouse line in which one endogenous allele of Cyp1a1 gene was replaced with the Cre gene, offering the potential for sensitive, tissue-specific control of Cre recombinase expression and therefore POR deletion. This allows Cre to be driven by the endogenous Cyp1a1 promoter (Cre^*Cyp1a1* − *KI*^). By crossing these with our original *Por*^lox/lox^ animals, we generated a targeted conditional model of POR deletion (Endogenous Reductase Locus, ERL). In the present study, we describe the initial validation and characterization of the ERL mouse line, and demonstrate the effect of hepatic and intestinal deletion of POR on the *in vivo* pharmacokinetics of three therapeutic drugs.

## Experimental

### Reagents

All chemicals were purchased from Sigma Chemical, except where indicated.

### Production of transgenic mice

**Figure 1:**
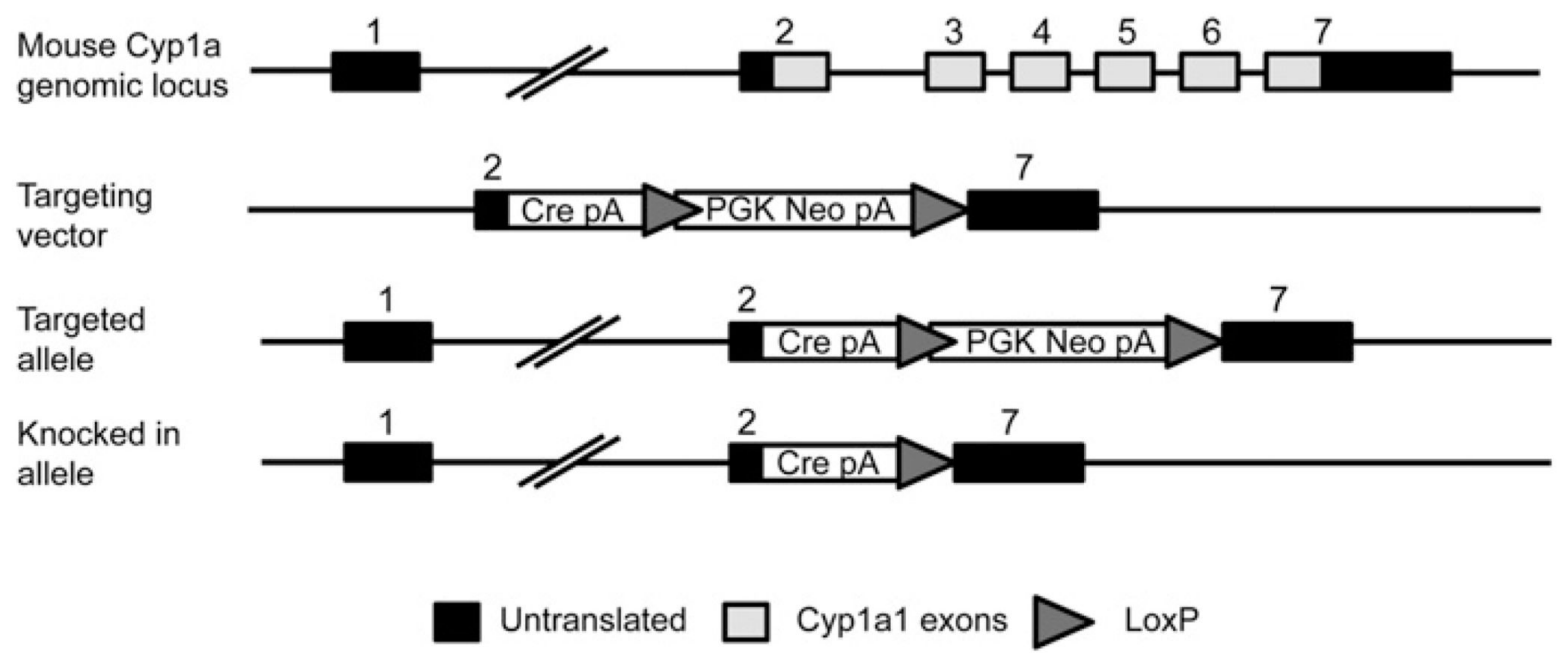
Targeting strategy for knockin of Cre recombinase to the murine Cyp1a1 locus: schematic representation of the murine Cyp1a1 locus Gene targeting was carried out in embryonic stem cells, replacing one copy of the murine Cyp1a1 gene with Cre recombinase (Cre pA). Cyp1a1 exons are shown as grey boxes and untranslated regions as black boxes. Triangles indicate the positions of the LoxP sites, which flank the neomycin cassette (Pgk Neo pA) and are used to delete the selectable marker in the final model. Cre^*Cyp1a1 − KI*^ mice were maintained by breeding with WT (C57BL/6N), and crossed on to floxed POR mice (*Por^lox/lox^*) to generate conditional inducible POR-null (Por^lox/lox^::Cre^*Cyp1a1 − KI*^) mice.

Por floxed mice [wild-type (WT): Por^lox/lox^] were generated as described previously [7]. Targeted insertion of Cre recombinase into the murine *Cyp1a1* locus of C57BL/6N ES cells was undertaken by TaconicArtemis, as described in Figure 1. Briefly, those parts of the murine Cyp1a1 gene which are translated – parts of exons 2 and 7, and all of exons 3–6 – were replaced by Cre recombinase, including a polyA sequence for transcript termination. Selection of correctly targeted clones was achieved by inclusion of a PGK-Neomycin cassette, flanked by FRT sites for subsequent removal *in vivo* by crossing on to a ubiquitously active general Flp deleter mouse, CAGGS-Flpe (Taconic Model 7089). The resulting mouse line (last line, Figure 1) was crossed on to the floxed POR line (Por^lox/lox^) to generate the ERL mouse (Por^lox/lox^::Cre^*Cyp1a1* − *KI*^). The mouse colony was maintained by breeding WT (Por^lox/lox^) with ERL animals (Por^lox/lox^/Cre^*Cyp1a1* − *KI*^), providing littermate controls for all experiments. All mice were maintained under standard animal house conditions with a 12-h light/12-h dark cycle and free access to water and RM1 diet (SDS). All studies were carried out on adult male or female mice (8–12 weeks old) in accordance with the Animal Scientific Procedures Act (1986) and after local ethical review.

### Mouse treatments

Single doses of *β*NF (80 mg/kg body weight as a suspension in corn oil) were administered intraperitoneally (i.p.). Doses of 3MC between 0.5 and 40 mg/kg body weight (single injections) were administered i.p. in corn oil; in some experiments, three daily doses were administered, as detailed in the Figure legends. Mice were killed at the same time of day by a rising concentration of CO_2_ 9 or 14 days after dosing and the following tissues removed and snap-frozen in liquid nitrogen: liver, lung, kidney, small intestine, large intestine, spleen and skin. Samples were then stored at − 80°C until processing for sub-cellular fractions.

### Immunoblotting and biochemical analysis

Microsomal fractions were prepared as described previously [25] and stored at − 70°C until required. Microsomal protein concentrations were determined using the Bio-Rad Protein Assay Reagent (Bio-Rad Laboratories) and Western blot analysis was preformed as described previously [26] using polyclonal antisera to rat P450s and POR [27,28]. Immunoreactive proteins were detected using horseradish peroxidase (HRP)-conjugated polyclonal goat anti-rabbit, anti-mouse or anti-sheep immunoglobulin secondary antibodies, as appropriate (Dako). Bands were visualized using Immobilon™ chemiluminescent HRP-conjugated substrate (Millipore) and a FUJIFILM LAS-3000 mini imaging system (Fujifilm UK.). Densitometric analysis was performed using Multi Gauge V2.2 software (Fujifilm UK).

### *In vivo* pharmacokinetics

WT and ERL mice (*n* = 3) were pre-treated with corn oil or 3MC (4 or 40 mg/kg). A single dose of midazolam (5 mg/kg p.o.), or nelfinavir (50 mg/kg p.o.) or paroxetine (10 mg/kg p.o.) was administered on day 14 after 3MC treatment. Whole blood (10 *μ*l) was taken from the tail vein at timed intervals after drug administration and transferred into a tube containing heparin (10 *μ*l, 15 IU/ml).

For midazolam analysis, internal standard solution (10 *μ*l; 500 ng of caffeine and 500 ng of resorpine) was added and proteins were precipitated using methanol (75 *μ*l), followed by 8% sulfosalicylic acid (55 *μ*l). Samples were mixed for 1 min and centrifuged at 13 000 ***g*** for 5 min. The supernatant was analysed for midazolam and the two internal standards by LC–MS/MS, as described previously [26]. Standard curves were constructed by spiking blank blood samples with known amounts of midazolam. Extraction and protein precipitation were carried out as outlined above for the test samples.

For nelfinavir and paroxetine analysis, mouse blood extraction was performed by a method based on protein precipitation using acetonitrile containing sulfadimethoxine (100 ng/ml) as internal standard. Samples were vortex mixed for 1 min followed by centrifugation at 2800 ***g*** for 10 min. Supernatants were analysed by UPLC–MS/MS using a Xevo TQs mass spectrometer (Waters). Nelfinavir, paroxetine and sulfadimethoxine were separated on an Acquity UPLC BEH C_18_ column (1.7 *μ*m particle size, 50 × 2.1 mm i.d.) by gradient elution with acetonitrile and water at a flow rate 0.6 ml/min. The cycle time of each analysis was 3 min. Matrix effects were assessed by dilution. Quantification of the analytes was performed in electrospray positive ionization mode using multiple reaction monitoring. The weighted (1/*x*^2^) calibration curve was linear over blood concentration range 1–1000 ng/ml with a correlation coefficient (*r*^2^) greater than 0.985 for every single analyte.

Fuller details of the analytical methods for nelfinavir and paroxetine are given in Supplementary information.

Pharmacokinetic parameters were calculated using WinNonLin software, version 3.1. A simple non-compartmental model was used to calculate the area under the curve (AUC), terminal half-life, *C*_max_ and clearance. Statistical analysis of pharmacokinetic data was carried out using Student’s *t* tests.

## Results and Discussion

The *Cre* gene was successfully targeted to the *Cyp1a1* locus in C57BL/6N ES cells using the strategy illustrated in Figure 1. The resulting mouse line was maintained in heterozygous form (i.e. *Cre^Cyp1a1 − KI/+^*) by crossing with WT C57BL/6N mice. The ERL line (Por^lox/lox^ ::Cre*^Cyp1a1 − KI^*) was generated by interbreeding *Cre^Cyp1a1 − KI/+^* and POR floxed mice [7]. In terms of gross phenotype, ERL animals were viable and physically normal. They were born at standard Mendelian ratios.

**Figure 2:**
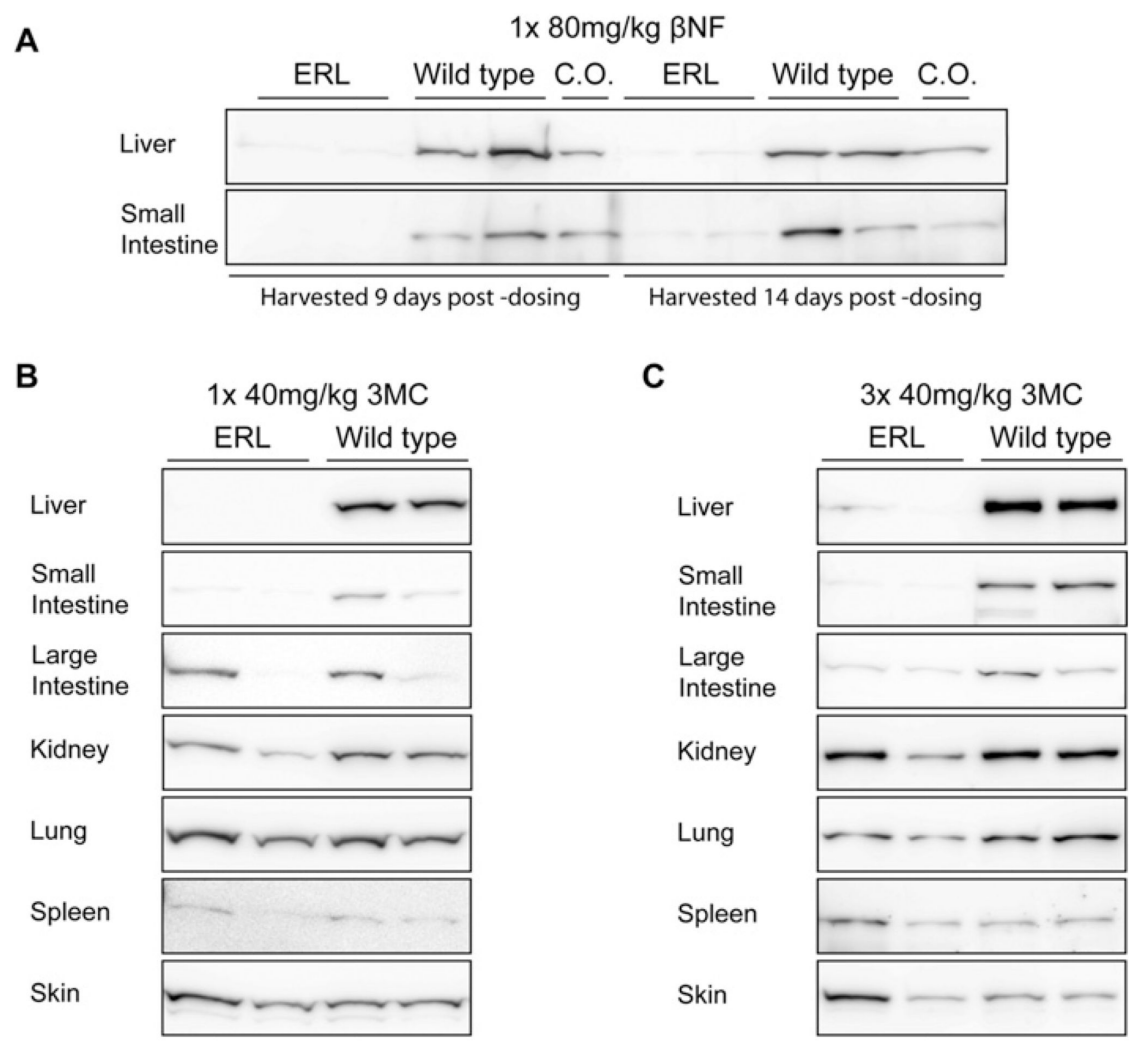
Deletion of P450 oxidoreductase with *β*-NF or 3-MC in various tissues (**A**) Western blots of POR expression in the liver and small intestine of ERL (Por^lox/lox^::Cre^*Cyp1a1 − KI*^) and WT (Por^lox/lox^) mice (*n* = 2) given a single injection of *β*NF (80 mg/kg i.p.). Tissues were harvested either 9 or 14 days later and individual microsomal fractions blotted as described. WT mice treated with corn oil (C.O.) only were used as vehicle control. (**B**) Western blots of POR expression in the liver, small intestine, lung, kidney, spleen and skin of ERL (Por^lox/lox^/Cre^*Cyp1a1 − KI*^) and WT (Por^lox/lox^) mice (*n* = 2) dosed with a single injection of 3MC (40 mg/kg i.p.). Tissues were harvested 14 days later, and individual microsomal fractions blotted as described. (**C**) Western blots of POR expression in the liver, small intestine, lung, kidney, spleen and skin of ERL (Por^lox/lox^::Cre^*Cyp1a1 − KI*^) and WT (Por^lox/lox^) mice (*n* = 2) dosed with 3MC (40 mg/kg i.p.) on three consecutive days. Tissues were harvested 14 days after the initial dose and individual microsomal fractions blotted as described.

The effects of a single dose of the polycyclic aromatic hydrocarbon AhR ligand *β*NF (80 mg/kg i.p.) on ERL mice were investigated in initial range-finding experiments (Figure 2A). Liver and small intestine tissues were harvested 9 or 14 days after dosing. The mice tolerated this dosing schedule reasonably well; although they tended to lose approximately 2–4 g in weight over the course of the treatment and occasionally exhibited signs of morbidity, this was less than in the previous (RCN) mouse model. On *post mortem* examination, *β*NF treated ERL mice had pale, mottled, enlarged livers which weighed approximately 60% more than those of either corn oil treated ERL mice or *β*NF treated WT animals. This phenotype is characteristic of POR deletion and is also observed in HRN and RCN animals [7,20]. The effect on POR expression in the ERL liver and small intestine of a single dose of *β*NF (80 mg/kg i.p.) was similar to that observed in RCN mice following four daily doses (compare Figure 2A with Figure 1 of Finn et al. [20]). No obvious phenotypic abnormalities were noted in the gross appearance of the small intestine.

A single dose of the AhR ligand 3MC (40 mg/kg i.p.) resulted in virtually complete elimination of POR expression in ERL liver and small intestine; however, POR expression in large intestine, lung, spleen, kidney and skin did not change in response to this treatment (Figures 2B and 2C). Both ERL and WT animals lost weight in response to this treatment. At *post mortem*, ERL mice were found to have enlarged, pale and mottled livers (60–90% increase in liver weight).

The effects of repeated dosing with 3MC on the deletion of POR were tested by administering three daily doses of 3MC (40 mg/kg/day i.p.) to ERL and WT mice. The effects of repeated dosing on the liver and intestine were similar to those of a single dose: POR was almost completely knocked down in the liver and small intestine and its expression was also reduced in the large intestine (50% knockdown); however, apart from a marginal reduction in POR expression in the lung after repeated doses of 3MC, no reduction in POR expression was observed in the other tissues examined (Figure 2C). Although residual hepatic POR expression was routinely determined to < 4% by densitometric quantification (results not shown), very faint bands were observed (Figures 2A and 2C), which may be related to length of exposure of the blots.

**Figure 3:**
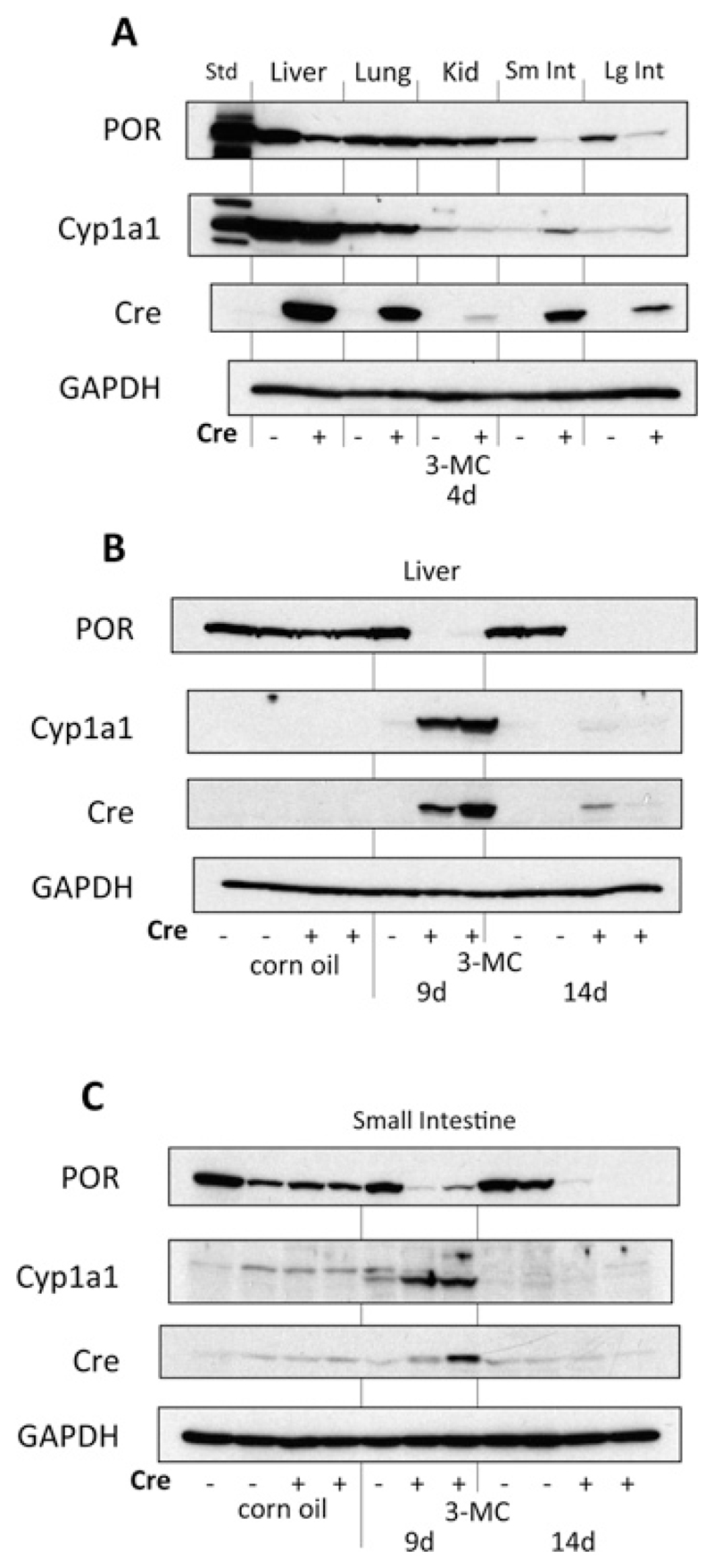
Time course of Cyp1a1 and Cre recombinase induction, and POR deletion in ERL mice following 3MC administration Adult male floxed POR mice without (−) or with Cre^*Cyp1a1 − KI*^ (+ ; ERL) were given a single dose of corn oil or 3MC (40 mg/kg i.p.) and harvested 4, 9 or 14 days later. Whole cell lysates (Cre) or microsomes (POR, Cyp1a1 and GAPDH) were prepared and analysed by immunoblotting as described. At day 4, samples represent pools of duplicate samples; at days 9 and 14, individual samples are shown. Std = recombinant POR or Cyp1a1 standard.

In order to establish an optimal dosing regimen for POR knockdown in the liver and small intestine, the effect of a single dose of 3MC was compared after 4, 9 and 14 days (Figure 3). At the 4-day time point, lung and kidney were also examined. In the liver, expression of POR was reduced but still readily detectable after 4 days, but was below the limit of immunochemical detection after 9 days. This was sustained at the 14 day time point (Figures 3A and 3B). Down-regulation appeared to occurmore quickly in the small intestine, being almost complete within 4 days (Figure 3A). Very low-level residual (or recovered) expression was detectable after nine and 14 days (Figures 3A and 3C). This may have been due to the more rapid turnover of gut epithelial cells compared with hepatocytes, and is consistent with previous observations in the RCN line as well as other lines in which intestinal gene deletion is driven by the AhCre construct [20,21].

The time-courses of induction of the endogenous Cyp1a1 protein and the product of the Cre transgene were also investigated (Figure 3). In the liver, intense expression of Cyp1a1 was observed 4 days after a single dose of 3MC and was sustained to the 9 day time-point but declined to background by day 14. The pattern of induction of the Cre protein in the liver was similar except that it was, of course, only present in animals carrying the Cre^Cyp1a1^ construct (Figures 3A and 3B). Induction of Cyp1a1 in the small intestine took a little longer, being barely detectable after 4 days, but also peaked after 9 days and declined by day 14 (Figures 3A and 3C). However, Cre induction in the small intestine happened more rapidly, being strongest after 4 days, declining slightly by day 9 and returning to baseline by day 14 (Figures 3A and 3C). As expected, this demonstrates that Cre induction precedes POR deletion but that POR deletion is sustained after the expression of Cre has returned to basal levels.

Comparison of the expression of POR, Cyp1a1 and Cre in different tissues (Figure 3A) illustrates that expression of Cre in a particular tissue is not necessarily predictive of POR deletion or Cyp1a1 induction, at least at the level of Western blot analysis of whole tissue lysates/microsomes. For example, marked upregulation of Cre occurs in the lung within 4 days of 3MC treatment (and is associated with intense expression of the endogenous Cyp1a1 protein) but is not associated with detectable down-regulation of POR. This may be a consequence of the fact that POR deletion only occurs in the small subpopulation of murine lung cells which respond to AhR ligands (Clara cells and/or Type II pneumocytes [29]), and it is more difficult to detect down-regulation in a small subpopulation of cells against strong background expression than to detect up-regulation against a low background. A similar explanation may account for the lack of detectable POR down-regulation in the kidney. A further possibility is that the half-life of POR may differ between tissues and under different cellular stress [30].Unfortunately,most papers reporting the use of the AhCre system to induce tissue-specific deletion of key genes do not identify the tissue types in which recombination does not occur, so it is difficult to evaluate these observations in the context of previous models.

**Figure 4:**
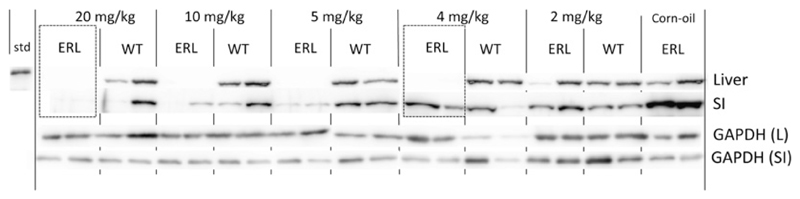
The effect on P450 oxidoreductase expression in the liver and small intestine of ERL (Por^lox/lox^/Cre^Cyp1a1 − KI^) and WT (Por^lox/lox^) mice of titrating the dose of 3MC Western blots of P450 oxidoreductase expression in the liver and small intestine (SI) of ERL (Por^lox/lox^::Cre^*Cyp1a1 − KI*^) and WT (Por^lox/lox^) in mice (*n* = 2) dosed with a single i.p. injection of 3MC (2, 4, 5, 10 or 20 mg/kg i.p.) and harvested 14 days later, as described. ERL mice treated with corn oil only were used as vehicle control. Std = recombinant POR; loading control was GAPDH. Boxes highlight POR deletion exclusively in liver at 4 mg/kg 3MC (right), and in both liver and small intestine at 20 mg/kg (left).

In order to characterize the dose–response for knockdown of POR in the liver and small intestine, the dose of 3MC administered was titrated between 0.5 and 20 mg/kg (single dose, i.p.) (Figure 4). In the liver, POR expression was reliably knocked down at doses above 4 mg/kg; at 2 mg/kg, POR was knocked down in one of the mice treated but not in the other. As with *β*NF, the dose of 3MC needed to knockdown POR in the liver of ERL mice was 10-fold lower than in RCN animals [20]. No down-regulation of intestinal POR expression was observed at 4 mg/kg; at 10 mg/kg, down-regulation occurred in one of two mice and a dose of 20 mg/kg led to POR down-regulation in both liver and gut. This compares favourably with the RCN model, in which no knockdown of small intestinal POR could be achieved with 3MC treatment even after repeated dosing. These data confirm that use of the endogenous *Cyp1a1* promoter to drive Cre recombinase expression generates an *in vivo* model that is far more sensitive to AhR ligands than the original RCN system.

**Figure 5:**
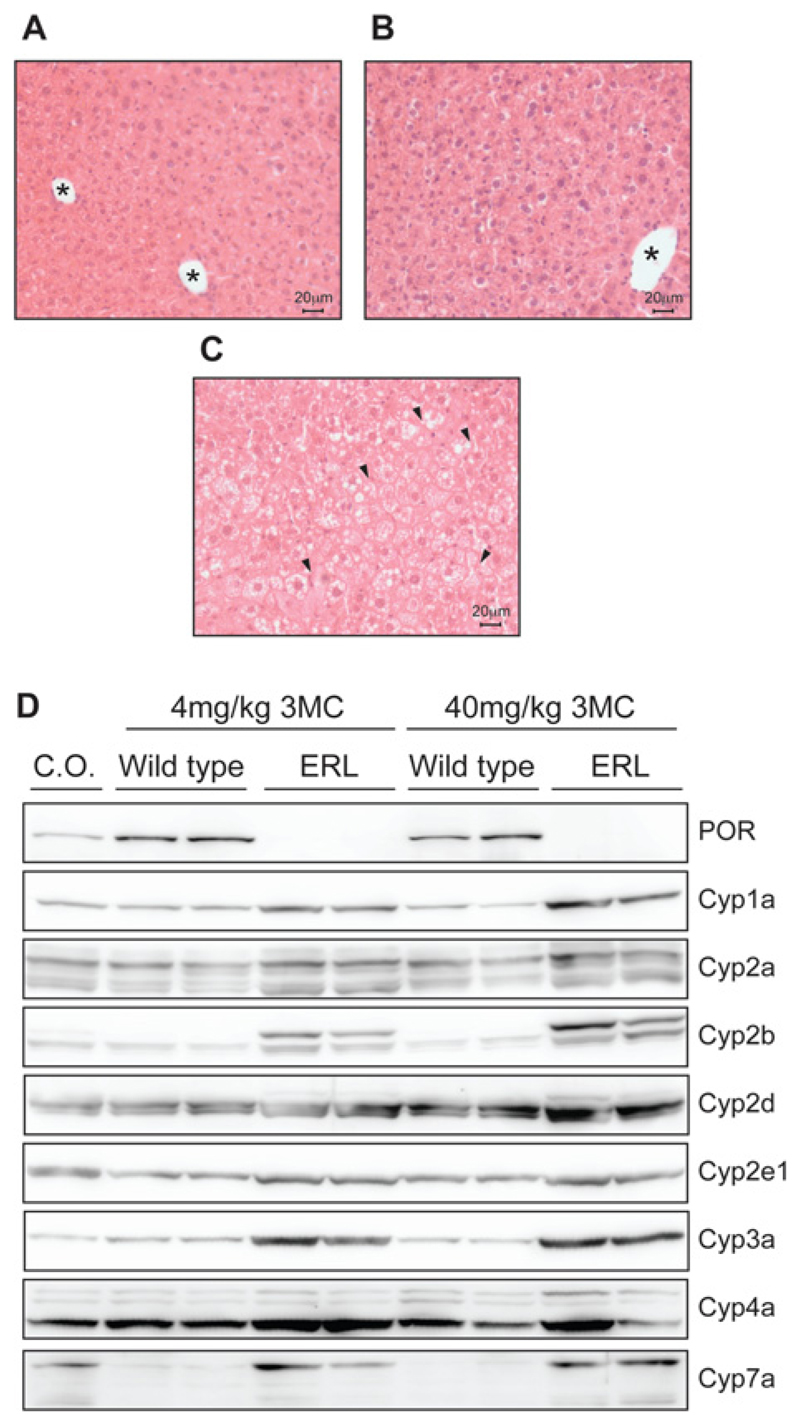
Histopathology and P450 expression in the livers of WT (Por^lox/lox^) and *ERL* (Por^lox/lox^/Cre^*Cyp1a1 − KI*^) mice Haematoxylin and eosin staining of liver sections from: (**A**) ERL (Por^lox/lox^/Cre^*Cyp1a1 − KI*^) treated with corn oil; (**B**) WT (Porlox/lox treated with 3MC (4 mg/kg); (**C**) ERL (Por^lox/lox^/Cre^*Cyp1a1 − KI*^) treated with 3MC (4 mg/kg). Arrowheads indicate vacuoles created by fat deposits; * designate blood vessels. (**D**) Western blot analysis of cytochrome P450 expression in the livers of WT (Por^lox/lox^) and ERL (Por^lox/lox^::Cre^*Cyp1a1 − KI*^) mice (*n* = 2) dosed with 3MC (4 or 40 mg/kg i.p.). WT mice treated with corn oil (C.O.) only were used as vehicle control.

One of the key phenotypes observed when POR is knocked down in the liver is hepatic steatosis resulting in increased hepatic P450 expression [7,8,20]. This up-regulation is caused, at least in part, by hepatic accumulation of unsaturated fatty acids which mediate up-regulation of P450 expression by activating the nuclear receptor CAR (constitutive androstane receptor) and, to a lesser degree, PXR (pregnane X receptor) [31]. As in the HRN and RCN models, haematoxylin and eosin staining of liver sections from ERL animals treated with 4 mg/kg 3MC exhibited the typical histology of a steatotic liver, with enlarged hepatocytes containing clear vacuoles typical of micro- and macro-vesicular hepatic lipidosis (Figures 5A–5C). Compensatory up-regulation of hepatic Cyp1a, Cyp2b, Cyp2c, Cyp2d, Cyp2e, Cyp3a, Cyp4a and Cyp7a was observed in ERL mice treated with either 4 or 40 mg/kg 3MC (Figure 5D), as observed previously in both the HRN and RCN models [7,20].

*In vitro* analysis of hepatic POR activity, measured using the surrogate electron acceptor cytochrome *c*, revealed that livers from ERL mice 14 days after treatment with 3MC at 40 mg/kg had approximately 4% of the activity of WT (2.2 ± 1.9 compared with 60.3 ± 20.8 nmol cytochrome *c* reduced/min/mg, *n* = 3, mean ± S.D., *P* < 0.01**).**This lack of POR expression in the liver translated into a 95% and 70% reduction in the P450-mediated turnover of 7-benzyloxy-4-(trifluoromethyl)-coumarin and methoxyresorufin (*n* = 6; *P* < 0.005 and *P* < 0.05, respectively). The low levels of cytochrome *c* reduction detected in the small intestine were further decreased (by 78%) in ERL animals treated with 40 mg/kg 3MC (36.2 ± 15.6 compared with 8.2 ± 3.3 nmol cytochrome *c* reduced/min/mg respectively, *n* = 3, mean + S.D., *P* < 0.01). No P450 activity was detected in small intestinal microsomes from either WT or ERL animals.

**Figure 6:**
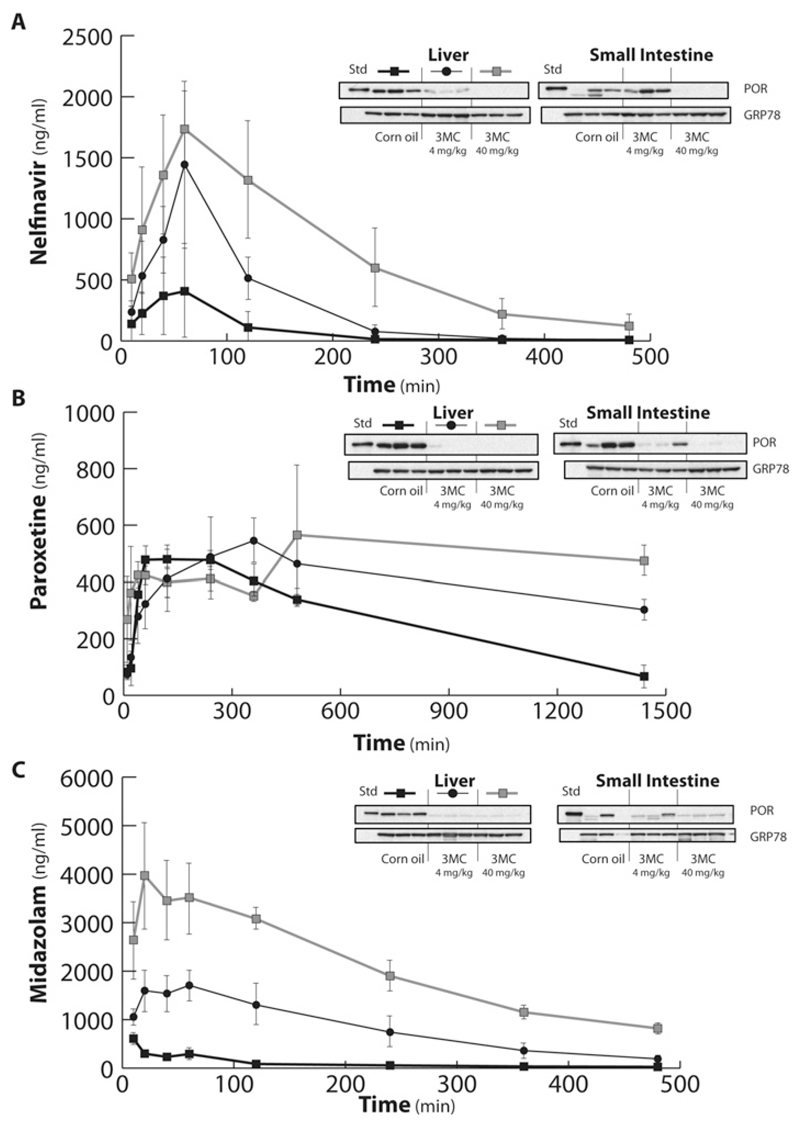
Effects of POR deletion in liver or liver and gut on blood concentrations of nelfinavir, paroxetine and midazolam WT and ERL mice (n = 3) were pre-treated with corn oil or 3MC (4 or 40 mg/kg), as described in the Experimental section. A single dose of nelfinavir (50 mg/kg p.o.), paroxetine (10 mg/kg p.o.) or midazolam (5 mg/kg p.o.) was administered on day 14 after 3MC treatment and a pharmacokinetic protocol carried out as described: (**A**) nelfinavir; (**B**) paroxetine; (**C**) midazolam. Black square, WT + Corn oil; black circle, ERL + 4 mg/kg 3MC (liver POR deletion); grey square, ERL + 40 mg/kg 3MC (liver and gut POR deletion). Pharmacokinetic analysis was conducted as described, data shown are means ± S.E.M. Inset: liver microsomal fractions were prepared and immunoblotted for POR and GRP78 (loading control) as described in the Experimental section.

In order to establish the functional consequences of POR deletion in liver or liver and small intestine, the pharmacokinetics of the P450 substrates nelfinavir, paroxetine and midazolam were investigated in ERL mice treated with doses of 3MC which would cause POR to be deleted either in the liver only (4 mg/kg) or in both the liver and intestine (40 mg/kg) (Figure 6 and Table 1). The HIV protease inhibitor nelfinavir is converted into a bioactive metabolite by CYP2C19 in humans [32], and deletion of POR in liver caused a marked increase in *C*_max_ and AUC (3.5- and 3.3-fold, respectively), and a decrease in clearance (84%). However, due to inter-animal variability these changes did not reach statistical significance and there was no change in half-life (Figure 6A and Table 1). However, when POR was deleted in both liver and gut, there were further increases in C_max_ and AUC for nelfinavir compared with WT mice (4.4- and 7.9-fold respectively), and an increased half-life (2.1-fold), all of which were statistically different from WT animals. Although clearance was further decreased from that for liver only POR deletion (by 93% relative to control) it did not achieve statistical significance. Western blotting for POR (Figure 6A, inset) confirmed deletion had occurred essentially as expected. In the case of the selective serotonin re-uptake inhibitor paroxetine, a substrate for several human P450 isoforms including CYP2C19 and CYP2D6 [33], a number of pharmacokinetic parameters were altered, although only AUC in the liver and gut POR deletion (1.5-fold increase), and clearance in the liver only POR deletion (~3-fold decrease), achieved statistical significance (Figure 6B and Table 1). C_max_ was essentially unchanged across all three models; although half-life was increased (3.5-fold) in the liver only POR deletion relative to control, this just failed to achieve statistical significance. It was not possible to calculate half-life or clearance for the liver and gut POR deletion as the pharmacokinetic data had not entered the terminal phase over the timeframe used in the experimental protocol. Western blotting showed that while POR deletion had occurred in both liver and gut at 40 mg/kg 3MC (Figure 6B, inset), POR deletion had also occurred to a significant extent in small intestine at the lower dose of 3MC used, thus making the interpretation of the role of gut metabolism in bioavailability difficult to ascertain. The sedative midazolam, a CYP3A4 substrate [34], showed statistically significant changes in all pharmacokinetic parameters except half-life for liver only POR deletion compared with WT, and for all parameters when comparing POR deletion in control or liver only to that in liver and gut (Figure 6C and Table 1). Deletion of POR in liver only led to a 3- and 9-fold increase in *C*_max_ and AUC, respectively, and a 9-fold decrease in clearance, relative to control, whereas the corresponding values for liver and gut POR deletion were 7.3- and 22.9-fold increases and a 26.3-fold decrease, respectively, with a 1.6-fold increase in half-life (Table 1). Between the liver only and liver and gut POR deletions, pharmacokinetic parameters were significantly changed, between 1.3- and 3-fold. Western blotting for POR, whereas variable for intestinal samples possibly due to difficulties in preparing microsomal fractions, showed deletion had generally occurred as predicted, suggesting that both liver and gut P450 metabolism contributed to midazolam disposition (Figure 6C, inset). These findings are consistent with the previously described role of hepatic and intestinal Cyp3a in midazolam clearance [35] and illustrate the importance of first pass metabolism in both the intestine and the liver in controlling peak concentrations of midazolam after oral dosing. They demonstrate the way in which the subtle manipulation of POR expression by use of different doses of 3MC can provide information about the relative importance of hepatic and intestinal P450-dependent metabolism in the metabolic clearance of drugs and other xenobiotics.

The model presented in the present paper represents a significant advance on our previous RCN model, which required the use of two different inducing agents, *β*NF and 3MC, to permit deletion of POR either in liver alone or liver plus intestine [20]. This model makes it much easier to achieve tissue-specific deletion of POR, simply by manipulating the dose of a single inducing agent. A particular value of this is that it allows direct comparison of hepatic and intestinal effects on drug and xenobiotic clearance by means of a simple dose–response experiment. In addition, it reduces the animal welfare issues associated with repeated administration of high doses of inducing agents, since it permits knockdown of hepatic POR expression with a single dose of 3MC at 4 mg/kg i.p., whereas the single dose required for knockdown in both liver and small intestine is 20–40 mg/kg i.p. This compares favourably with the doses required when randomly integrated AhCre is used to down-regulate gene expression. The regimens used for this purpose vary but usually involve repeated daily doses of *β*NF (typically 80 mg/kg i.p.) for up to 5 days, whereas one study has reported the administration of *β*NF at 160 mg/kg i.p. for 6 days [36], an exposure which is within the toxic range for this compound [37].

It should be noted that there was some inconsistency in the deletion of POR in the small intestine at 4 mg/kg 3MC based on Western blot analysis and rates of cytochrome c reduction. It is unclear to what extent this reflects difficulties in preparation of microsomal fractions from this tissue as opposed to inconsistency in the effectiveness of the inducing agent.

A further advantage of the ERL model is that the AhCre construct has been targeted to the endogenous Cyp1a1 locus rather than being allowed to integrate at random, as in conventional transgenesis. In conventional transgenics, the transgene integrates randomly at a single site in the genome. The location of integration cannot be predicted in advance, nor can the number of copies, which may range from one to as many as 50 in end-to-end (concatameric) orientation. This can lead to unexpected patterns of gene expression due to integration effects and/or to adverse phenotypic effects due to disruption of key endogenous genes. In contrast, the strategy adopted here located the Cre transgene in the appropriate genomic region for authentic control via the Cyp1a1 promoter, including both *cis* and *trans* regulatory mechanisms.

Despite the care taken to place the AhCre transgene in the appropriate genomic location, it was somewhat disappointing to observe that Cre induction by Ah ligands did not lead to down-regulation of POR in all the tissues where Cyp1a1 expression is subject to AhR regulation: in our experiments there was no evidence of POR deletion in the kidney, lung, spleen or skin. This may be a function of the method used to evaluate the knockdown (Western blotting of tissue lysates/microsomes), since effects occurring in small subpopulations of cells may have been obliterated by high level POR expression in non Ah-responsive cell types. Other possibilities are that the subpopulation of cells which expresses Cre following induction is not the same one in which POR is expressed, or that the subpopulation in which POR is knocked down has a very short lifespan and has already been replaced by new cells by the time analysis is conducted. Interestingly, collaborators using the *Cre^Cyp1a1 − KI^* model combined with a ROSA26-Lox-STOP-lox YFP system have found reporter expression in haematopoietic cells in the skin and lung, and in T lymphocytes from the intraepithelial and lamina propria layers of the gut (D.A. Cantrell and B. Stockinger, personal communication), demonstrating that the regulatable system works in a number of cells and tissues.

Other investigators have used the AhCre method to regulate the expression of a variety of genes, including *β*-catenin [21], E2f3 [38], Apc [39–41], Brca2 [42], K-ras [36,43,44], Pten [45], Lkb1 [46,47] and Ihh [48]. The main sites of induced recombination in these models are usually the crypts and villi of the small intestine [21,36,38,42–45,47–49]. In general, these studies have focused on key genes involved in cell cycle regulation and carcinogenesis in the intestine, and have therefore not addressed other tissue types; however, interesting observations have been reported in some other organs and cell types following dosing with AhR ligands. For example, when the AhCre system was used to introduce constitutively expressed mutant K-ras genes by deletion of a floxed STOP cassette in C57BL/6 mice, *β*NF-induced recombination was observed in the large intestine, caecum, stomach, spleen, liver, pancreas, kidney and lung [36,43]. Furthermore, tissue-specific reporter expression (i.e. Cre-mediated deletion of a STOP cassette) was observed in the small intestine, liver, bladder, pancreas and interfollicular epithelial cells of the skin when the AhCre^ER^ dual control system was used to drive expression of the ROSA26-LacZ and ROSA26-LacZ/EYFP reporter in the absence of background expression [50,51]. In addition, the AhCre system has been used to achieve tissue-specific knockdown of Lkb1 expression in the genitourinary tract [46]. The conventional AhCre system has the limitation that background recombination can occur in, for example, the liver, kidneys, bladder, oesophagus and cardiac muscle, even in the absence of a xenobiotic inducer [50]; however, some investigators have made use of this background recombination, for example, to study the effects of oncogene mutation and tumour suppressor gene deletion on renal carcinogenesis [44,47,52].

In summary, we have generated a mouse model in which a gene target – in the present study, POR – may be deleted in a conditional, inducible manner; the ERL mouse represents a significant improvement over our previous RCN model. Furthermore, the simplicity of use of the ERL model provides the opportunity for further manipulation of the tissue-specific expression of POR by the use of alternative dosing routes (e.g. topical application for knockdown in the skin) and evaluation of additional AhR ligands as tissue or cell-type specific tools for POR (or any floxed gene locus) regulation.

## Supplementary Material

Supporting Information

## Figures and Tables

**Table 1:** Effects of POR deletion in liver or liver and gut on the pharmacokinetics of nelfinavir, paroxetine and midazolam WT and ERL mice were pre-treated with corn oil or 3MC (4 mg/kg, liver POR deletion; 40 mg/kg, gut POR deletion). A single dose of nelfinavir (50 mg/kg), paroxetine (10 mg/kg p.o.) or midazolam (5 mg/kg p.o.) was administered on day 14 after 3MC treatment. Data are means ± S.D.; pharmacokinetic analysis was conducted as described and data compared using a Student’s *t* test. *P < 0.05; **P < 0.01; ***P < 0.005: control compared with liver null; ^•^ *P* < 0.05; ^••^ *P* < 0.01; ^•••^ *P* < 0.005: control compared with liver and gut null; ^◦^ *P* < 0.05; liver compared with liver and gut null.

Drug	Half-life (min)	*C*_max_ (*μ*g/ml)	AUC_all_ (min *μ*g/ml)	Cl (ml/min/kg)
Nelfinavir				
Control	49 ± 8	0.42 ± 0.48	43 ± 48	2348 ± 1722
Liver null	51 ± 10	1.4 ± 0.84	142 ± 51	377 ± 116
Liver and gut null	104 ± 45^••^	1.8 ± 0.18^•^	339 ± 134^•^	164 ± 91
Paroxetine				
Control	447 ± 136	0.5 ± 0.02	392 ± 51	23 ± 4.7
Liver null	1565 ± 727	0.56 ± 0.13	578 ± 131	8.3 ± 2.6**
Liver and gut null	†	0.5 ± 0.04	594 ± 46^••^	†
Midazolam				
Control	109 ± 24	0.61 ± 0.11	43 ± 12	113 ± 35
Liver null	134 ± 17	1.85 ± 0.34***	395 ± 141*	13 ± 5**
Liver and gut null	180 ± 16^•◦^	4.46 ± 1.53^• ◦^	988 ± 251^•••◦^	4.3 ± 0.9^••◦^

† Pharmacokinetic data did not reach terminal phase.

## Abbreviations

AhR: aryl hydrocarbon receptor
*β*NF: *β*-naphthoflavone
ERL: Endogenous Reductase Locus
HRP: horseradish peroxidase
HRN: Hepatic Reductase Null
PhIP: 2-amino-1-methyl-6-phenylimidazo[4,5-*b*]pyridine
POR: cytochrome P450 reductase
P450: cytochrome P450
3MC: 3-methylcholanthrene
RCN: Reductase Conditional Null

## References

[1] Riddick DS, Ding X, Wolf CR, Porter TD, Pandey AV, Zhang QY, Gu J, Finn RD, Ronseaux S, McLaughlin LA (2013). NADPH-cytochrome P450 oxidoreductase: roles in physiology, pharmacology, and toxicology. Drug Metab Dispos.

[2] Emoto C, Iwasaki K (2007). Approach to predict the contribution of cytochrome P450 enzymes to drug metabolism in the early drug-discovery stage: the effect of the expression of cytochrome b(5) with recombinant P450 enzymes. Xenobiotica.

[3] Emoto C, Murayama N, Rostami-Hodjegan A, Yamazaki H (2010). Methodologies for investigating drug metabolism at the early drug discovery stage: prediction of hepatic drug clearance and P450 contribution. Curr Drug Metab.

[4] Otto DM, Henderson CJ, Carrie D, Davey M, Gundersen TE, Blomhoff R, Adams RH, Tickle C, Wolf CR (2003). Identification of novel roles of the cytochrome P450 system in early embryogenesis: effects on vasculogenesis and retinoic Acid homeostasis. Mol Cell Biol.

[5] Schmidt K, Hughes C, Chudek JA, Goodyear SR, Aspden RM, Talbot R, Gundersen TE, Blomhoff R, Henderson C, Wolf CR, Tickle C (2009). Cholesterol metabolism: the main pathway acting downstream of cytochrome P450 oxidoreductase in skeletal development of the limb. Mol Cell Biol.

[6] Shen AL, O’Leary KA, Kasper CB (2002). Association of multiple developmental defects and embryonic lethality with loss of microsomal NADPH-cytochrome P450 oxidoreductase. J Biol Chem.

[7] Henderson CJ, Otto DM, Carrie D, Magnuson MA, McLaren AW, Rosewell I, Wolf CR (2003). Inactivation of the hepatic cytochrome P450 system by conditional deletion of hepatic cytochrome P450 reductase. J Biol Chem.

[8] Gu J, Weng Y, Zhang QY, Cui H, Behr M, Wu L, Yang W, Zhang L, Ding X (2003). Liver-specific deletion of the NADPH-cytochrome P450 reductase gene: impact on plasma cholesterol homeostasis and the function and regulation of microsomal cytochrome P450 and heme oxygenase. J Biol Chem.

[9] Wu L, Gu J, Weng Y, Kluetzman K, Swiatek P, Behr M, Zhang QY, Zhuo X, Xie Q, Ding X (2003). Conditional knockout of the mouse NADPH-cytochrome p450 reductase gene. Genesis.

[10] Arlt VM, Stiborova M, Henderson CJ, Osborne MR, Bieler CA, Frei E, Martinek V, Sopko B, Wolf CR, Schmeiser HH, Phillips DH (2005). Environmental pollutant and potent mutagen 3-nitrobenzanthrone forms DNA adducts after reduction by NAD(P)H:quinone oxidoreductase and conjugation by acetyltransferases and sulfotransferases in human hepatic cytosols. Cancer Res.

[11] Pass GJ, Carrie D, Boylan M, Lorimore S, Wright E, Houston B, Henderson CJ, Wolf CR (2005). Role of hepatic cytochrome p450s in the pharmacokinetics and toxicity of cyclophosphamide: studies with the hepatic cytochrome p450 reductase null mouse. Cancer Res.

[12] Arlt VM, Stiborova M, Henderson CJ, Thiemann M, Frei E, Aimova D, Singh R, Gamboa da Costa G, Schmitz OJ, Farmer PB (2008). Metabolic activation of benzo[a]pyrene *in vitro* by hepatic cytochrome P450 contrasts with detoxification *in vivo*: experiments with hepatic cytochrome P450 reductase null mice. Carcinogenesis.

[13] Henderson CJ, Pass GJ, Wolf CR (2006). The hepatic cytochrome P450 reductase null mouse as a tool to identify a successful candidate entity. Toxicol Lett.

[14] Levova K, Moserova M, Kotrbova V, Sulc M, Henderson CJ, Wolf CR, Phillips DH, Frei E, Schmeiser HH, Mares J (2011). Role of cytochromes P450 1A1/2 in detoxication and activation of carcinogenic aristolochic acid I: studies with the hepatic NADPH:cytochrome P450 reductase null (HRN) mouse model. Toxicol Sci.

[15] Stiborova M, Arlt VM, Henderson CJ, Wolf CR, Kotrbova V, Moserova M, Hudecek J, Phillips DH, Frei E (2008). Role of hepatic cytochromes P450 in bioactivation of the anticancer drug ellipticine: studies with the hepatic NADPH: cytochrome P450 reductase null mouse. Toxicol Appl Pharmacol.

[16] Fang C, Gu J, Xie F, Behr M, Yang W, Abel ED, Ding X (2008). Deletion of the NADPH-cytochrome P450 reductase gene in cardiomyocytes does not protect mice against doxorubicin-mediated acute cardiac toxicity. Drug Metab Dispos.

[17] Conroy JL, Fang C, Gu J, Zeitlin SO, Yang W, Yang J, VanAlstine MA, Nalwalk JW, Albrecht PJ, Mazurkiewicz JE (2010). Opioids activate brain analgesic circuits through cytochrome P450/epoxygenase signaling. Nat Neurosci.

[18] Zhang QY, Fang C, Zhang J, Dunbar D, Kaminsky L, Ding X (2009). An intestinal epithelium-specific cytochrome P450 (P450) reductase-knockout mouse model: direct evidence for a role of intestinal p450s in first-pass clearance of oral nifedipine. Drug Metab Dispos.

[19] Lin Y, Yao Y, Liu S, Wang L, Moorthy B, Xiong D, Cheng T, Ding X, Gu J (2012). Role of mammary epithelial and stromal P450 enzymes in the clearance and metabolic activation of 7,12-dimethylbenz(a)anthracene in mice. Toxicol Lett.

[20] Finn RD, McLaren AW, Carrie D, Henderson CJ, Wolf CR (2007). Conditional deletion of cytochrome P450 oxidoreductase in the liver and gastrointestinal tract: a new model for studying the functions of the P450 system. J Pharmacol Exp Ther.

[21] Ireland H, Kemp R, Houghton C, Howard L, Clarke AR, Sansom OJ, Winton DJ (2004). Inducible Cre-mediated control of gene expression in the murine gastrointestinal tract: effect of loss of beta-catenin. Gastroenterology.

[22] Campbell SJ, Carlotti F, Hall PA, Clark AJ, Wolf CR (1996). Regulation of the CYP1A1 promoter in transgenic mice: an exquisitely sensitive on-off system for cell specific gene regulation. J Cell Sci.

[23] Campbell SJ, Henderson CJ, Anthony DC, Davidson D, Clark AJ, Wolf CR (2005). The murine Cyp1a1 gene is expressed in a restricted spatial and temporal pattern during embryonic development. J Biol Chem.

[24] Arlt VM, Singh R, Stiborova M, Gamboa da Costa G, Frei E, Evans JD, Farmer PB, Wolf CR, Henderson CJ, Phillips DH (2011). Effect of hepatic cytochrome P450 (P450) oxidoreductase deficiency on 2-amino-1-methyl-6-phenylimidazo[4,5-b]pyridine-DNA adduct formation in P450 reductase conditional null mice. Drug Metab Dispos.

[25] Meehan RR, Forrester LM, Stevenson K, Hastie ND, Buchmann A, Kunz HW, Wolf CR (1988). Regulation of phenobarbital-inducible cytochrome P-450s in rat and mouse liver following dexamethasone administration and hypophysectomy. Biochem J.

[26] Finn RD, McLaughlin LA, Ronseaux S, Rosewell I, Houston JB, Henderson CJ, Wolf CR (2008). Defining the *in vivo* role for cytochrome b5 in cytochrome P450 function through the conditional hepatic deletion of microsomal cytochrome b5. J Biol Chem.

[27] Forrester LM, Henderson CJ, Glancey MJ, Back DJ, Park BK, Ball SE, Kitteringham NR, McLaren AW, Miles JS, Skett P (1992). Relative expression of cytochrome P450 isoenzymes in human liver and association with the metabolism of drugs and xenobiotics. Biochem J.

[28] Smith GC, Tew DG, Wolf CR (1994). Dissection of NADPH-cytochrome P450 oxidoreductase into distinct functional domains. Proc Natl Acad Sci USA.

[29] Hynes DE, DeNicola DB, Carlson GP (1999). Metabolism of styrene by mouse and rat isolated lung cells. Toxicol Sci.

[30] Liu D, Waxman DJ (2002). Post-transcriptional regulation of hepatic NADPH-cytochrome P450 reductase by thyroid hormone: independent effects on poly(A) tail length and mRNA stability. Mol Pharmacol.

[31] Finn RD, Henderson CJ, Scott CL, Wolf CR (2009). Unsaturated fatty acid regulation of cytochrome P450 expression via a CAR-dependent pathway. Biochem J.

[32] Hirani VN, Raucy JL, Lasker JM (2004). Conversion of the HIV protease inhibitor nelfinavir to a bioactive metabolite by human liver CYP2C19. Drug Metab Dispos.

[33] Jornil J, Jensen KG, Larsen F, Linnet K (2010). Identification of cytochrome P450 isoforms involved in the metabolism of paroxetine and estimation of their importance for human paroxetine metabolism using a population-based simulator. Drug Metab Dispos.

[34] van Herwaarden AE, Smit JW, Sparidans RW, Wagenaar E, van der Kruijssen CM, Schellens JH, Beijnen JH, Schinkel AH (2005). Midazolam and cyclosporin a metabolism in transgenic mice with liver-specific expression of human CYP3A4. Drug Metab Dispos.

[35] van Waterschoot RA, van Herwaarden AE, Lagas JS, Sparidans RW, Wagenaar E, van der Kruijssen CM, Goldstein JA, Zeldin DC, Beijnen JH, Schinkel AH (2008). Midazolam metabolism in cytochrome P450 3A knockout mice can be attributed to up-regulated CYP2C enzymes. Mol Pharmacol.

[36] Luo F, Brooks DG, Ye H, Hamoudi R, Poulogiannis G, Patek CE, Winton DJ, Arends MJ (2009). Mutated K-ras(Asp12) promotes tumourigenesis in Apc(Min) mice more in the large than the small intestines, with synergistic effects between K-ras and Wnt pathways. Int J Exp Pathol.

[37] McKillop D, Case DE (1991). Mutagenicity, carcinogenicity and toxicity of beta-naphthoflavone, a potent inducer of P448. Biochem Pharmacol.

[38] Chong JL, Wenzel PL, Saenz-Robles MT, Nair V, Ferrey A, Hagan JP, Gomez YM, Sharma N, Chen HZ, Ouseph M (2009). E2f1–3 switch from activators in progenitor cells to repressors in differentiating cells. Nature.

[39] Sansom OJ, Reed KR, Hayes AJ, Ireland H, Brinkmann H, Newton IP, Batlle E, Simon-Assmann P, Clevers H, Nathke IS (2004). Loss of Apc *in vivo* immediately perturbs Wnt signaling, differentiation, and migration. Genes Dev.

[40] Sansom OJ, Reed KR, van de Wetering M, Muncan V, Winton DJ, Clevers H, Clarke AR (2005). Cyclin D1 is not an immediate target of beta-catenin following Apc loss in the intestine. J Biol Chem.

[41] March HN, Rust AG, Wright NA, ten Hoeve J, de Ridder J, Eldridge M, van der Weyden L, Berns A, Gadiot J, Uren A (2011). Insertional mutagenesis identifies multiple networks of cooperating genes driving intestinal tumorigenesis. Nat Genet.

[42] Hay T, Patrick T, Winton D, Sansom OJ, Clarke AR (2005). Brca2 deficiency in the murine small intestine sensitizes to p53-dependent apoptosis and leads to the spontaneous deletion of stem cells. Oncogene.

[43] Luo F, Brooks DG, Ye H, Hamoudi R, Poulogiannis G, Patek CE, Winton DJ, Arends MJ (2007). Conditional expression of mutated K-ras accelerates intestinal tumorigenesis in Msh2-deficient mice. Oncogene.

[44] Sansom OJ, Meniel V, Wilkins JA, Cole AM, Oien KA, Marsh V, Jamieson TJ, Guerra C, Ashton GH, Barbacid M, Clarke AR (2006). Loss of Apc allows phenotypic manifestation of the transforming properties of an endogenous K-ras oncogene *in vivo*. Proc Natl Acad Sci USA.

[45] Marsh V, Winton DJ, Williams GT, Dubois N, Trumpp A, Sansom OJ, Clarke AR (2008). Epithelial Pten is dispensable for intestinal homeostasis but suppresses adenoma development and progression after Apc mutation. Nat Genet.

[46] Pearson HB, McCarthy A, Collins CM, Ashworth A, Clarke AR (2008). Lkb1 deficiency causes prostate neoplasia in the mouse. Cancer Res.

[47] Shorning BY, Zabkiewicz J, McCarthy A, Pearson HB, Winton DJ, Sansom OJ, Ashworth A, Clarke AR (2009). Lkb1 deficiency alters goblet and paneth cell differentiation in the small intestine. PLoS One.

[48] van Dop WA, Heijmans J, Buller NV, Snoek SA, Rosekrans SL, Wassenberg EA, van den Bergh Weerman MA, Lanske B, Clarke AR, Winton DJ (2010). Loss of Indian Hedgehog activates multiple aspects of a wound healing response in the mouse intestine. Gastroenterology.

[49] Buczacki SJ, Zecchini HI, Nicholson AM, Russell R, Vermeulen L, Kemp R, Winton DJ (2013). Intestinal label-retaining cells are secretory precursors expressing Lgr5. Nature.

[50] Kemp R, Ireland H, Clayton E, Houghton C, Howard L, Winton DJ (2004). Elimination of background recombination: somatic induction of Cre by combined transcriptional regulation and hormone binding affinity. Nucleic Acids Res.

[51] Clayton E, Doupe DP, Klein AM, Winton DJ, Simons BD, Jones PH (2007). A single type of progenitor cell maintains normal epidermis. Nature.

[52] Sansom OJ, Griffiths DF, Reed KR, Winton DJ, Clarke AR (2005). Apc deficiency predisposes to renal carcinoma in the mouse. Oncogene.

