# Supplementary material for: Application of a novel regulatable Cre recombinase system to define the role of liver and gut metabolism in drug oral bioavailability
Source: Biochem J. Author manuscript; Available in PMC 2020 Jan 8. (PMC6949133; doi:10.1042/BJ20140582)
Supplement: Supporting Information [file EMS85291-supplement-Supporting_Information.pdf]

Supplemental Information

UPLC-MS/MS analysis – paroxetine and nelfinavir

Standard stock solutions, calibration standards and quality control samples

Individual stock solutions (1mg/mL) of nelfinavir, paroxetine and sulfadimethoxine (internal standard) were prepared by dissolving the accurately weighted reference standard in dimethylsulphoxide (DMSO).

Calibration line and quality control (QC) samples were prepared by spiking 95µL of diluted blood with 5µL of the appropriate daughter solution. Calibration curve standards were made at concentrations of 1, 2, 4, 10, 20, 40, 100, 200, 400, 1000ng/mL whereas high, medium and low QC samples were prepared at 100, 40 and 2ng/mL, respectively.

Blood sample preparation

All study samples were collected per time point by venepuncture and serial bleeding into tubes containing an equal volume of water/heparin solution and stored in the freezer. On the day of analysis samples were thawed at room temperature before extraction.

Internal standard solution was added to all calibration samples, QCs and blanks and samples vortexed. Dilution in acetonitrile containing internal standard was 3:1 for all samples.

All samples were then centrifuged for 10 min at 2,800 rpm. Supernatant from study samples and calibration samples, QCs, double blanks and blanks was then transferred to wells of a 96 well plate for analysis.

Liquid chromatography and mass spectrometric settings

Samples were analysed by an Acquity UPLC System coupled to a Xevo TQs mass spectrometer (Waters, USA). Chromatographic analysis was conducted on a Acquity UPLC BEH C18 analytical column (50x2.1mm, 1.7μm; Waters, USA) maintained at 40°C in a column oven. The mobile phase consisted of water with 0.01% formic acid and acetonitrile with 0.01% formic acid and it was delivered at a flow rate of 0.6mL/min under gradient conditions.

Ionization and detection was conducted on a Xevo™ TQs mass spectrometer (Waters, USA) equipped with a ScanWave™ technology operating in a positive electrospray ionisation mode.

Multiple reaction monitoring (MRM) was carried out using precursor → product ion transitions of *m/z* nelfinavir 568.29→134.69, paroxetine 330.15→192.05, and sulfadimethoxine 311.52→155.91.

The source-dependent parameters maintained for all analytes and internal standard were as follows: capillary (kV), 0.5; Cone (V), 3.0; desolvation temperature (°C), 600; desolvation gas flow (L/hr), 1000; collision gas flow (mL/min), 0.15. The foremost values for compound parameters like cone voltage and collision energy were 16 and 47 for nelfinavir, 60 and 19 for paroxetine and 16 and 40 for sulfadimethoxine, respectively. Quadrupoles 1 and 3 were maintained at unit mass resolution and the dwell time was set at 0.045ms requiring a minimum of 15 points per peak for all test-compounds. Data collection, peak integration and calculations were performed using Mass Lynx software, version 2.0.
